# ZASC1 Stimulates HIV-1 Transcription Elongation by Recruiting P-TEFb and TAT to the LTR Promoter

**DOI:** 10.1371/journal.ppat.1003712

**Published:** 2013-10-24

**Authors:** James W. Bruce, Rachel Reddington, Elizabeth Mathieu, Megan Bracken, John A. T. Young, Paul Ahlquist

**Affiliations:** 1 Morgridge Institute for Research, Madison, Wisconsin, United States of America; 2 Institute for Molecular Virology, University of Wisconsin, Madison, Wisconsin, United States of America; 3 McArdle Laboratory for Cancer Research, University of Wisconsin, Madison, Wisconsin, United States of America; 4 Howard Hughes Medical Institute, University of Wisconsin, Madison, Wisconsin, United States of America; 5 Nomis Foundation Laboratories for Immunobiology and Microbial Pathogenesis, The Salk Institute for Biological Studies, La Jolla, California, United States of America; University of Pennsylvania School of Medicine, United States of America

## Abstract

Transcription from the HIV-1 LTR promoter efficiently initiates but rapidly terminates because of a non-processive form of RNA polymerase II. This premature termination is overcome by assembly of an HIV-1 TAT/P-TEFb complex at the transactivation response region (TAR), a structured RNA element encoded by the first 59 nt of HIV-1 mRNA. Here we have identified a conserved DNA-binding element for the cellular transcription factor, ZASC1, in the HIV-1 core promoter immediately upstream of TAR. We show that ZASC1 interacts with TAT and P-TEFb, co-operating with TAT to regulate HIV-1 gene expression, and promoting HIV-1 transcriptional elongation. The importance of ZASC1 to HIV-1 transcription elongation was confirmed through mutagenesis of the ZASC1 binding sites in the LTR promoter, shRNAs targeting ZASC1 and expression of dominant negative ZASC1. Chromatin immunoprecipitation analysis revealed that ZASC1 recruits Tat and P-TEFb to the HIV-1 core promoter in a TAR-independent manner. Thus, we have identified ZASC1 as novel regulator of HIV-1 gene expression that functions through the DNA-dependent, RNA-independent recruitment of TAT/P-TEFb to the HIV-1 promoter.

## Author Summary

The human immunodeficiency virus 1 (HIV-1) promoter exhibits a strong block to transcription elongation that is a critical regulator of the viral life cycle. Failure to overcome this restriction during provirus establishment results in a transcriptionally silent, latent provirus. Similarly, reactivation from this latent state requires overcoming this block to transcription elongation. The virus achieves this through the concerted action of TAR, a structured RNA element in the nascent transcript, and the virally encoded transactivator of transcription (TAT) protein. TAT bound to TAR stimulates transcription by interacting with and activating the cellular elongation factor P-TEFb. Once activated P-TEFb is released from a repressive complex, it stimulates transcription elongation by phosphorylating the C-terminal domain of RNA polymerase II. Here we describe the identification of ZASC1 as a novel activator of TAT-mediated gene expression. ZASC1 is a transcription factor with strong links to cancer and inherited ataxias. We identified highly conserved ZASC1 binding sites in the HIV-1 promoter and demonstrate that ZASC1 stimulates TAT transcription elongation. We show that ZASC1 complexes with and recruits both TAT and P-TEFb to the HIV-1 LTR promoter in a TAR-independent, DNA-dependent manner, defining a new step in HIV-1 transcription activation.

## Introduction

The *Retroviridae* family includes human immunodeficiency viruses type-1 and 2 (HIV-1 and HIV-2), the causative agents of acquired immune deficiency syndrome (AIDS). Retroviruses are unique among RNA viruses in that after virus entry into the cell, the viral RNA is reverse transcribed into double stranded DNA and integrated into the cellular chromosome, generating the provirus. This feature makes retroviruses dependent on the host RNA polymerase II transcription machinery for expressing viral gene products and new genomes.

Transcription of integrated proviral DNA is driven from the unique 3′ (U3) element in the viral genome. This is a strong RNA polymerase II (pol II) promoter that contains many overlapping binding sites for cellular transcription factors that modulate expression in different cell types and response to signaling pathways [1], [2]. In addition, HIV-1 transcription is regulated by the viral TAT protein. In the absence of TAT, transcription is efficiently initiated, but low levels of full-length transcripts are produced from the HIV-1 LTR promoter due to stalled pol II [3]. TAT overcomes this block by recruiting the cellular transcriptional elongation factor P-TEFb to the transactivation response region (TAR), a structured RNA element located from +1 to +59 in nascent HIV-1 mRNA. Subsequent phosphorylation of the negative elongation factor (NELF), the SUPT5 component of DSIF and the C-terminal domain (CTD) of pol II by P-TEFb results in release of stalled polymerase, transfer of TAT/P-TEFb to the extending polymerase and a dramatic increase in transcription elongation [4], [5].

P-TEFb is a heterodimer of cyclin T1 (CycT1) and cyclin dependent kinase (Cdk9) [6]. The majority of P-TEFb is maintained in cells in an inactive state, bound to the 7SK snRNP, a complex of 7SK snRNA and the Larp7, Mepce, and HEXIM1 or 2 proteins [7], [8], [9], [10]. Active P-TEFb is released from the inhibitory 7SK snRNA through the action of signal transduction pathways or by TAT [5]. The mechanism and location of P-TEFb extraction from 7SK snRNP by TAT remain controversial. However, it is clear that multiple interactions probably facilitate the mobilization of free P-TEFb, including: high affinity interactions between CycT1 and TAT [11], [12], [13], [14], competition between TAT and HEXIM1 for the binding domain on the 7SK RNA [15], and competitive displacement the 7SK RNA from P-TEFb by the TAR element [16]. In addition to interacting with the TAR element, several reports suggest that optimal TAT function requires specific sequences flanking the HIV-1 core promoter around the TATA box, potentially through interactions with cellular DNA binding proteins [16], [17], [18], [19], [20], [21], [22], [23].

Cellular transcription factors regulate not only HIV LTR-driven gene expression during productive infection, but also the switch to a transcriptionally inactive state of the provirus known as latency [24], [25]. Latently infected cells serve as a reservoir of infected cells that avoid highly active anti-retroviral therapy (HAART), which targets viral enzymes. Subsequent reactivation of virus production from latently infected cells prevents patients from clearing the virus and cycling off the HAART drugs [26]. Understanding the role of cellular transcription factors during productive and latent proviral expression could lead to new therapies that inhibit reactivation or stimulate reactivation for subsequent clearing of latently infected cells by HAART.

Previously we reported a genetic screen of insertionally mutagenized Chinese hamster ovary (CHO-K1) cells that identified cellular transcription factor ZASC1 as a novel regulator of MLV transcription [27]. The *ZASC1* gene encodes a protein with nine Kruppel-like zinc fingers with broad tissue distribution [28]. ZASC1 copy number amplification is linked to multiple squamous cell carcinomas and an increased propensity for metastasis [28], [29], [30]. Furthermore, ZASC1 interacts with histone acetyltransferase CBP [31], is involved in **β**-catenin nuclear transport [32] and has been associated with inherited ataxias [33].

We recently showed that ZASC1 is a sequence-specific DNA binding protein with three highly similar binding sites in the MLV U3 promoter [27]. Here we show that the HIV-1 promoter contains a highly conserved ZASC1 binding site (ZBS) located just upstream of the TAR element. The data presented below further reveal that ZASC1 binds to specific DNA elements in the HIV-1 LTR and regulates proviral transcription by stimulating HIV-1 TAT activity. Furthermore, we found that ZASC1 recruits TAT and P-TEFb to the HIV-1 promoter in the presence and absence of TAR. Thus, we have identified a new, RNA-independent, DNA-dependent step in the recruitment of TAT/P-TEFb to the HIV-1 promoter, and demonstrated that ZASC1 is a critical factor in the regulation of HIV-1 transcription elongation.

## Results

### ZASC1 binds to the HIV-1 promoter

**Figure 1:**
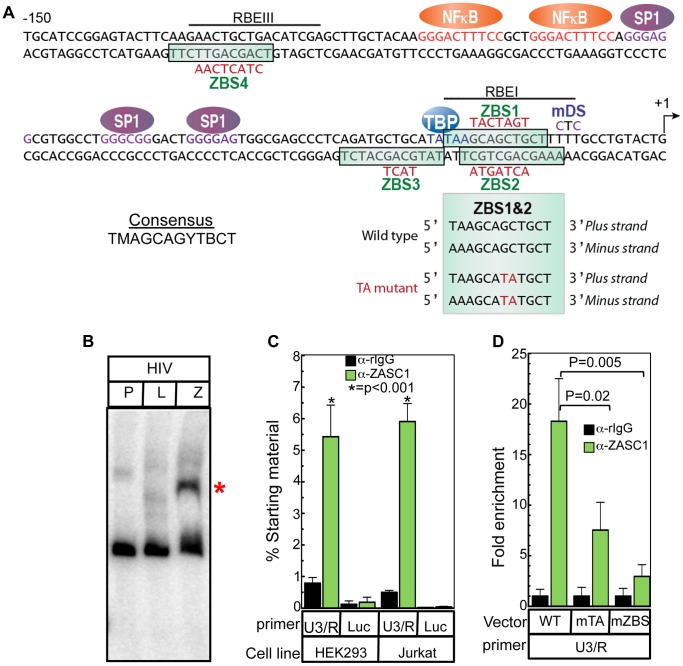
ZASC1 binds to specific DNA elements in the HIV-1 promoter and regulates viral transcription. (A) Sequence of the HIV U3 region from −150 to +1 of HIV-1 isolate NL43 showing the relative location of the four putative ZBS. Known transcription factor binding sites are indicated as follows: NFκB (orange), SP1 (purple), TBP (blue) and the Ras-responsive binding elements (RBE) RBEI and RBEIII (underlined). Mutations that were introduced in the ZBS are indicated in red. A control downstream mutation (mDS) is indicated in purple. Alignment of ZBS 1 and 2 indicating the 2 bp offset palindromic structure and the GC to TA mutation introduced into the site that alters the same bases on both strands is shown below the promoter sequence. (B) EMSA of the WT HIV U3 DNA probe (nucleotides −454 to +66) alone (P) or incubated with in vitro transcribed/translated luciferase (L) or ZASC1 (Z) proteins. (C) Total ZASC1 bound to the HIV promoter was measured by chromatin immunoprecipitation with an anti-ZASC1 antibody on HEK293 or Jurkat cells transduced with NL43E-R-Luc using a primer set that spans the ZBS at the U3/R boundary (−116 to +25) or a primer that amplifies the luciferase gene in the nef locus located approximately 9 kBp downstream from the transcription start site. Real-time PCR analysis was performed in triplicate and normalized to input controls. (D) ChIP assays of ZASC1 recruitment to the HIV-1 promoter in Jurkat cells transduced with similar levels of NL43E-R-Luc or modified viral constructs containing the TA mutation or the mutation of all four ZBS (Fig. 1A). Immunoprecipitation and Real-time PCR analysis was performed in triplicate and normalized to input controls and is reported as fold-enrichment relative to control pull-downs with non-specific rabbit IgG. The data shown are the average mean values obtained in an experiment performed with quadruplicate samples and each is representative of three independent experiments. Error bars indicate the standard deviation of the data in all panels. P-values were calculated using a standard Student's t-test and significant changes relative to WT or relevant bracketed comparisons indicated.

The consensus ZASC1 DNA binding site (ZBS) (TMAGCAGYTBCT) that we previously identified in the MLV promoter [27] was used to search for similar related sequences in other retroviral promoters. This analysis revealed four sequences in the HIV-1 U3 promoter similar to the consensus MLV ZBS (Fig. 1A). The four potential ZASC binding sites (ZBS 1 through 4) in HIV-1 align well with those in MLV and each contain at most two bases that diverge from the MLV consensus ZBS. Consistent with this, a specific high molecular weight mobility shift was observed when ZASC1 was incubated with an HIV-1 U3 DNA fragment in an *in vitro* EMSA experiment but not when the same fragment was incubated with a control firefly luciferase protein (Fig. 1B). These results demonstrate that ZASC1 can bind specifically to the HIV-1 promoter *in vitro*.

To confirm that ZASC1 is bound to the putative ZASC1 binding sites in the HIV-1 promoter in infected cells, we performed chromatin immunoprecipitation (ChIP) experiments from HEK293 (human embryonic kidney cells) and Jurkat (a human T-cell line) cells challenged with the HIV-1 vector, NL43E-R-Luc. This vector is a full length proviral clone containing stop mutations in the VPR and ENV genes and it has a WT LTR promoter, encodes TAT, and contains a firefly luciferase reporter gene in place of NEF [34]. Immunoprecipitation with an anti-ZASC1 antibody enriched the U3/R boundary region (nt −116 to +25) of the HIV-1 promoter by 6.9- and 11.8-fold in chromatin from HEK293 and Jurkat cells, respectively, relative to a non-specific IgG control (Fig. 1C). The primer set used to amplify this region spanned the start site of transcription and ZBS 1, 2, and 3 (Fig. 1B). As expected, no enrichment was observed with primer sets that amplified the luciferase gene, which is located approximately 9 kb downstream of the transcription start site (Fig. 1C). Similarly, the anti-ZASC1 antibody only precipitated DNA fragments located within 500 bp of the transcription start site (data not shown).

To determine the effect of ZBS mutations on recruitment of ZASC1 to the HIV-1 promoter, mutations of all four putative ZBS (mZBS) were introduced into NL43-E-R-luc (Fig. 1A). Additionally we noted that ZBS 1 and 2 (ZBS1,2) overlap and form a two bp offset inverted palindrome that partially overlaps the TATA box (Fig. 1A). To more specifically target these palindromic sites and avoid disrupting the TATA box, a two bp mutation was also introduced into ZBS1,2 resulting in equivalent base pairs in the ZBS on each strand from GC to TA (mTA) (green box, Fig. 1A). Jurkat cells were challenged with WT NL43-E-R-luc, mTA or the mZBS variant. ChIP assays revealed that the TA mutation significantly reduced the ZASC1 enrichment from 18.3- fold to 7.5-fold, while the more severe mutation reduced ZASC1 enrichment to 3- fold (Fig. 1D). Thus, while the TA mutation significantly impairs ZASC1 binding, mutation of all four ZBS's severely inhibits ZASC1 recruitment to the HIV-1 promoter.

**Figure 2:**
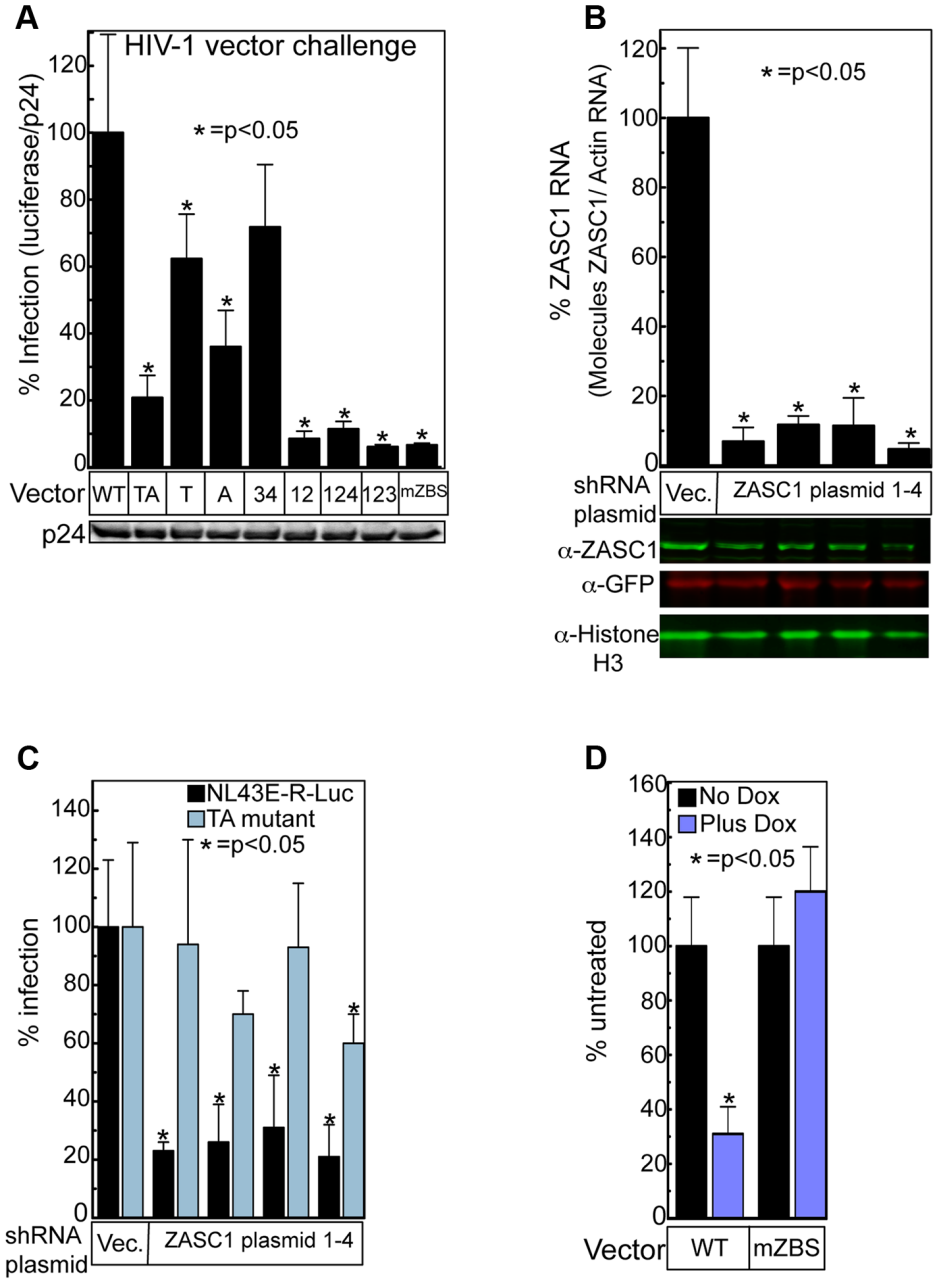
ZASC1 contributes to HIV-1 vector gene expression. (A) Jurkat cells were challenged in quadruplicate with NL43E-R-Luc derivatives containing mutations in the indicated ZBS as described in Fig. 1A. Infections were monitored by chemiluminescent assays as described in materials and methods and normalized to input virus by anti-capsid (p24) western blots and reported as the ratio of [reporter gene activity∶input capsid] observed. (B) HEK293T (2×10^5^ cell/well of 12 well plate) cells were transiently transfected with 1 µg of plasmids expressing GFP and either one of four shRNAs targeting the open reading frame of ZASC1 or the empty shRNA vector. Three days post transfection, replicate wells were harvested for either total RNA or protein. Quantitative RT-PCR analysis of ZASC1 and actin RNA was performed in triplicate (Bar graph), representative western blots with the indicated antibody are shown below. (C) HEK293T cells (1×10^4^ cells/well) in a 96 well plate were transiently transfected with 80 ng of plasmids expressing either one of four shRNAs targeting the open reading frame of ZASC1 or the empty shRNA vector along with an 20 ng of an expression vector encoding the ASLV receptor TVA800 [35], [36]. Three days post transfection, cells were challenged with EnvA pseudotyped HIV vectors, either NL43E-R-Luc, which directs HIV LTR-driven firefly luciferase gene expression, or the mTA mutant (Fig. 1A). (D) HeLa cells expressing the Tet repressor were stably transduced with a lentivirus construct encoding a Tet-inducible dominant negative GFP-ZASC1 fusion protein. GFP-ZASC1 expression was induced with 1 µg/ml doxycycline, 24 hours post induction the cells were challenged with the VSV-G pseudotyped NL43E-R-Luc or a derivative with all 4 ZBS mutated (Fig. 1A). Infection was monitored using chemiluminescent assays as described in materials and methods. The data shown are the average mean values obtained in an experiment performed with quadruplicate samples and each is representative of three independent experiments. Error bars indicate the standard deviation of the data in all panels. P-values were calculated using a standard Student's t-test and significant changes relative to WT indicated.

Viruses harboring mutations in all four ZBSs were shown to be defective for virus replication in human Jurkat T cells as judged by luciferase reporter expression (Fig. 2A). While mutation of HIV ZBS3 and 4 did not significantly impair luciferase expression, mutation of the overlapping binding sites 1 and 2 resulted in a 10-fold reduction (Fig. 2A). Therefore, the palindromic ZBS sites located immediately downstream of the TATA box are important for efficient virus gene expression following infection. Consistent with this, the mTA variant exhibited a 5-fold reduction in reporter gene expression when compared to the WT promoter construct, and even single nucleotide substitutions in these binding sites (T or A) showed a significant reduction in reporter gene expression (Fig. 2A). Importantly, introduction of these mutations into the 3′ LTR did not affect virus yield in producer cells, as indicated by an anti-P24 western blot of undiluted viral stocks used for the infection (Fig. 2A, blot under graph). Taken together, these data indicate that ZASC1 is recruited to the palindromic ZBS1,2 sites, strongly contributing to HIV-1 gene expression.

### shRNA depletion of ZASC1 inhibits HIV-1 infection

The role of ZASC1 in HIV-1 gene expression was further validated by transient RNAi-mediated knockdown of ZASC1 in human HEK293T cells. These cells were transiently transfected with plasmids encoding shRNAs that were previously shown to specifically target ZASC1 [27], along with an expression vector encoding the avian sarcoma and leukosis virus (ASLV) receptor TVA800. Under these conditions, there was a 10- to 20-fold reduction in ZASC1 mRNA and a 2- to 3-fold reduction in ZASC1 protein expression (Fig. 2B). Three days post transfection, the cells were infected with either WT NL43E-R-Luc or the mTA mutant viruses pseudotyped with the ASLV subgroup A envelope protein (EnvA). Since mammalian cells do not express the receptor for EnvA [35], [36], only cells co-transfected with both TVA-800 and the shRNAs were infected by the pseudotyped viruses. Independent expression of four shRNAs targeting different regions of the ZASC1 mRNA inhibited reporter gene expression from WT NL43E-R-Luc vector 3.2- to 4.8-fold (Fig. 2C). In stark contrast, these shRNAs did little to inhibit expression from the vector with the ZBS TA mutation (1.1- to 1.7-fold reduction), ruling out that the effects were due to non-specific or off-target effects, and demonstrating an important role for ZASC1 in gene expression from the HIV-1 LTR promoter.

### Dominant negative ZASC1 inhibits HIV-1 expression

We previously demonstrated that a GFP-ZASC1 fusion protein exhibits dominant negative activity toward WT ZASC1 function [27]. To determine if this GFP-ZASC1 fusion protein could also inhibit HIV vector expression, we employed a tetracycline-inducible system. HeLa-Trex cells (Invitrogen), which express the Tet-repressor, were transduced with a lentiviral vector that encodes the GFP-ZASC1 fusion protein under the control of a tetracycline-inducible promoter. GFP-ZASC1 expression was induced with doxycycline and, 48 hours post induction, the cells were challenged with VSV-G pseudotyped NL43E-R-Luc derivatives with either a WT or mZBS LTR promoter (see Fig. 1A). After dox treatment, reporter gene expression from the WT vector was reduced by 3.2-fold (Fig. 2D). In contrast, dox treatment had no significant effect on the mutant vector (Fig. 2D). Thus, mutating the putative ZBS (Fig. 2A) or interfering with ZASC1 function by either shRNA-mediated knockdown (Fig. 2C) or through expression of a dominant negative form of ZASC1 (Fig. 2D), decreased gene expression from the HIV-1 promoter.

### ZASC1 affects transcription elongation from the HIV-1 promoter

**Figure 3:**
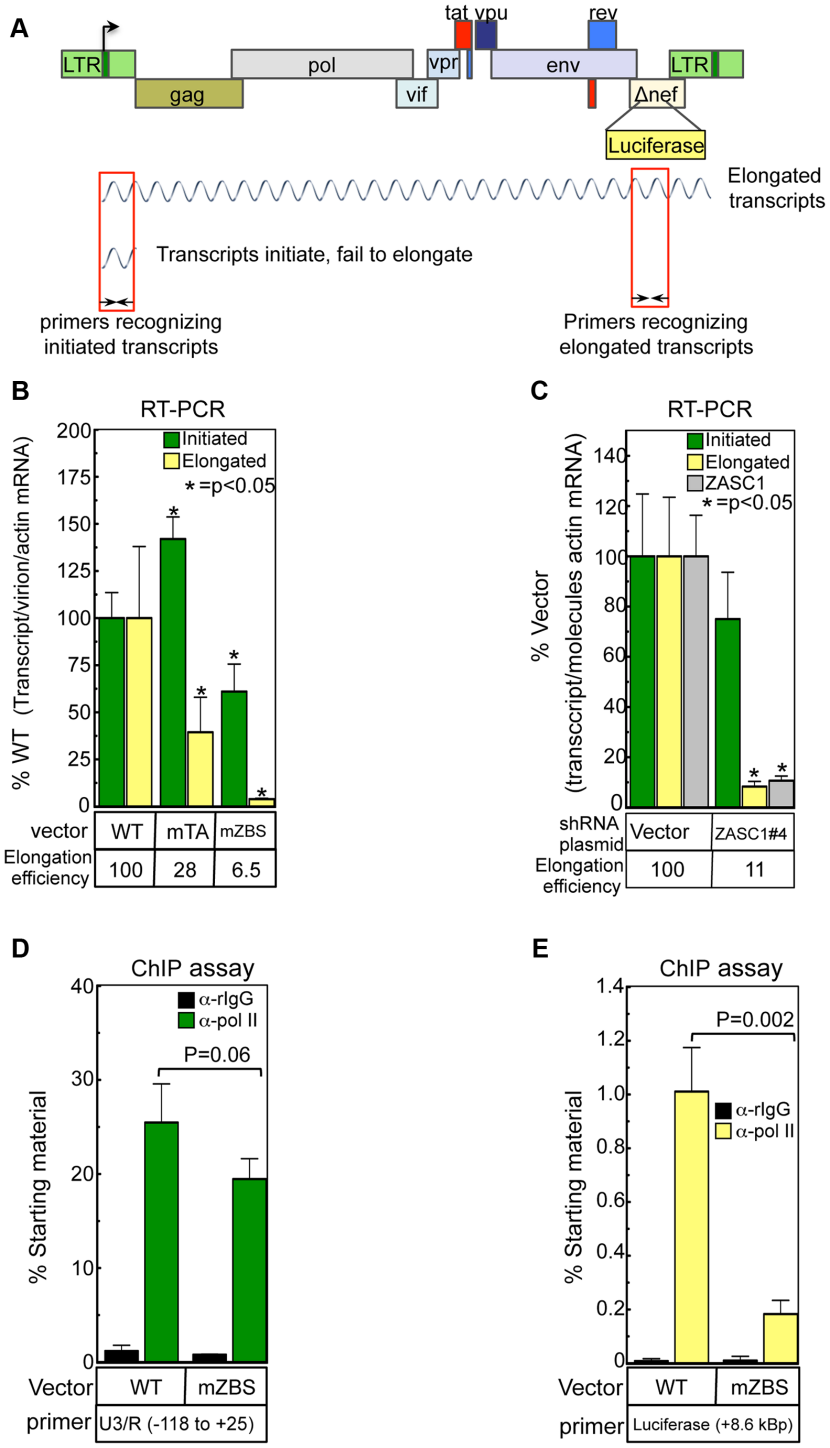
ZASC1 promotes HIV-1 transcription elongation. (A) Schematic of the HIV-1 vector NL43E-R-Luc indicating the two types of primary transcripts and the PCR primers utilized to differentiate them. (B) The amount of initiated and extended transcripts was assayed in cells challenge with NL43E-R-Luc or ZBS variants by isolating total RNA 48 hpi and performing real-time reverse transcription PCR with primers that amplify all initiated transcripts (+1 to +58) or transcripts that have been initiated and extended (+9961 to +10,191). Transcript values were normalized for the amount of input virion RNA and total cellular actin mRNA. Real-time PCR was performed in triplicate. Elongation efficiency of panel B was determined by dividing the extended transcripts by the total initiated transcripts with WT efficiency set at 100%. (C) HEK293 cells (1×10^5^) were transfected with an shRNA vector plasmid or an shRNA targeting ZASC1 (shRNA ZASC1#4, see Fig. 2B), 72 hrs post transfection, the cells were challenged with WT NL43-luc. 48 hrs post infection, total RNA was harvested and initiated and extended transcripts were analyzed and elongation efficiency determined as in B. ZASC1 RNA was analyzed as in Fig. 2B. (D) Total RNA polymerase II bound to either NL43E-R-Luc or a derivative with all ZBS mutated (Fig. 1A) was measured by chromatin immunoprecipitation assays using a primer set that spans the transcription initiation site (−116 to +25) or (E) a primer set that amplifies the luciferase gene in the nef locus approximately 9 kBp downstream from the transcription start site. Error bars indicate the standard deviation of the data and are representative of three independent experiments. P-values were calculated using a standard Student's t-test and significant changes relative to WT or relevant bracketed comparisons indicated.

ZASC1 binding may regulate HIV-1 gene expression by promoting transcription initiation or elongation from the HIV-1 promoter (Fig. 3A). To determine if transcription initiation, elongation or both are affected by ZASC1, we isolated RNA from virus-infected cells and used quantitative PCR analysis to compare the levels of total initiated transcripts with a primer set that amplifies the first 58 nucleotides of all viral transcripts, including those generated by RNA pausing in the absence of TAT [37]. Levels of successfully elongated transcripts were measured with a primer set that amplifies the luciferase transgene inserted into the *nef* locus 9 kBp downstream of the transcription start site (Table 1 and Fig. 3A). This luciferase amplicon was chosen because its level would be expected to correlate well with the luciferase expression by HIV-1 vectors. The amount of initiated and extended viral transcripts were measured and reported as the ratio of viral transcript/input p24/cellular actin transcript. The viral stocks used in these extension experiments varied by less than 2-fold from WT. Since actin levels and input titer change little, this is essentially a measure of initial expression per input virion. The amounts of initiated and extended transcripts observed in WT vector infections were set at 100%. RNA was isolated 48 hours after challenge with equivalent amounts of WT NL43-luc, mTA, or mZBS and assayed by quantitative real-time RT-PCR. The altered mTA virus (Fig. 1A) exhibited a slight, but reproducible, increase in initiated transcripts, possibly due to an increased TA content in the core promoter [38], but reduced extension (2.6-fold) relative to the virus with the wild-type promoter (Fig. 3B). If the increased initiation efficiency is taken into consideration, by dividing the %elongated by the % initiated (i.e. elongation efficiency), the impairment in extension rises to a 3.6-fold difference (Fig. 3B, elongation efficiency, below graph). The mZBS virus exhibited a slight (39%) decrease in initiation, but a 25-fold decrease in elongation (Fig. 3B). Correcting for the slight defect in elongation results in an elongation efficiency of 15.4-fold for the mZBS mutation (Fig. 3B). These data correlate well with the observed reduction in reporter gene expression (Fig. 2A).

**Table 1:** Real-time PCR primers.

Target gene^a^	Oligo Name	orientation^b^	Sequence 5′ to 3′^c^	Binding position^d^
HIV U3/R	OJWB904	sense	GAGCTTGCTACAAGGGACTTTC	−116
	OJWB809	antisense	CAGGCTCAGATCTGGTCTAAC	25
fLuc	OJWB891	sense	TGTACACGTTCGTCACATCTCATC	9291
	OJWB892	antisense	TTATCAGTGCAATTGTTTTGTCACG	9386
gLuc	OJWB820	sense	AGTGTTCTGACCTGCTCAAGA	536
	OJWB821	antisense	AGTCACCACCGGCCCCCTTGA	637
HIV-R	OJWB694	sense	GGGTCTCTCTGGTTAGACCAG	1
	OJWB695	antisense	GGGTTCCCTAGTTAGCCAGAG	58
gag	OJWB872	sense	CGAGGATTGTGGAACTTCTGGGACGC	8556
	OJWB873	antisense	TTATAGCAAAATCCTTTCCAAGCCCTGTC	8791
fLUC	OJWB874	sense	AGGCGAATTATGTGTCAGAGGACCTATG	9961
	OJWB875	antisense	GTTGTAACAATATCGATTCCAATTCAGCG	10191
vif	OJWB910	sense	ATGGCAGGTGATGATTGTGTG	5052
	OJWB911	antisense	CCATGTGTTAATCCTCATCCTGTC	5103
ZASC1	OJWB1005	sense	CTACCTGGTTCCCAGTCCTGTA	NA
	OJWB1006	antisense	GCTGGTTGACTGATTCAGGTGAG	
Actin	OJWB887	sense	TCACCCACACTGTGCCCATCTACGA	NA
	OJWB888	antisense	CAGCGGAACCGCTCATTGCCAATGG	

*^a^*The gene or viral component that the primer amplifies.

*^b^*Denotes whether the oligo binds on the coding strand (relative to the mRNA) or noncoding strand.

*^c^*Sequence of the oligo written in the 5′ to 3′ orientation.

*^d^*The region of amplification, relative to the HIV-1 transcription start site in construct.

To confirm that the defect in elongation was due to the functions of ZASC1 and not an artifact of the LTR mutations, we transfected cells with the most effective ZASC1 shRNA plasmid (shRNA ZASC#4, Fig. 2B) and assayed for HIV-1 initiated and elongated transcripts. Under conditions in which ZASC1 RNA was knocked down 9.3-fold (Fig. 3C, grey bar), initiated transcripts were reduced by only 25%, but elongated transcripts were impaired 12-fold (Fig. 3C). After correcting for the slight effect on elongation, ZASC1 knockdown affected elongation efficiency by 9-fold (Fig. 3C). Thus, shRNA knockdown of ZASC1 phenotypically copies the effect of the ZBS mutations. Together, these results demonstrate that the primary action of ZASC1 on the HIV-1 promoter is to promote transcription elongation.

Consistent with a specific role for ZASC1 in transcription elongation, ChIP assays revealed similar enrichment of RNA polymerase II at the transcription initiation site of proviruses with either a WT LTR promoter or the mutant mZBS promoter (Fig. 3D). Indeed, the mutated sites exhibited slightly greater enrichment than the WT promoter (25- vs. 22- fold, respectively). By contrast, similar ChIP assays revealed over 5-fold less RNA polymerase II associated with the downstream luciferase reporter gene in the mZBS versus WT viral constructs (Fig. 3E). This effect correlates well with the strong reduction in HIV-1-directed reporter activity observed in vector challenge assays with this ZBS variant (Fig. 2A) and the reduction in transcription elongation products (Fig. 3B).

### ZASC1 regulates transcription elongation in primary T-cells

**Figure 4:**
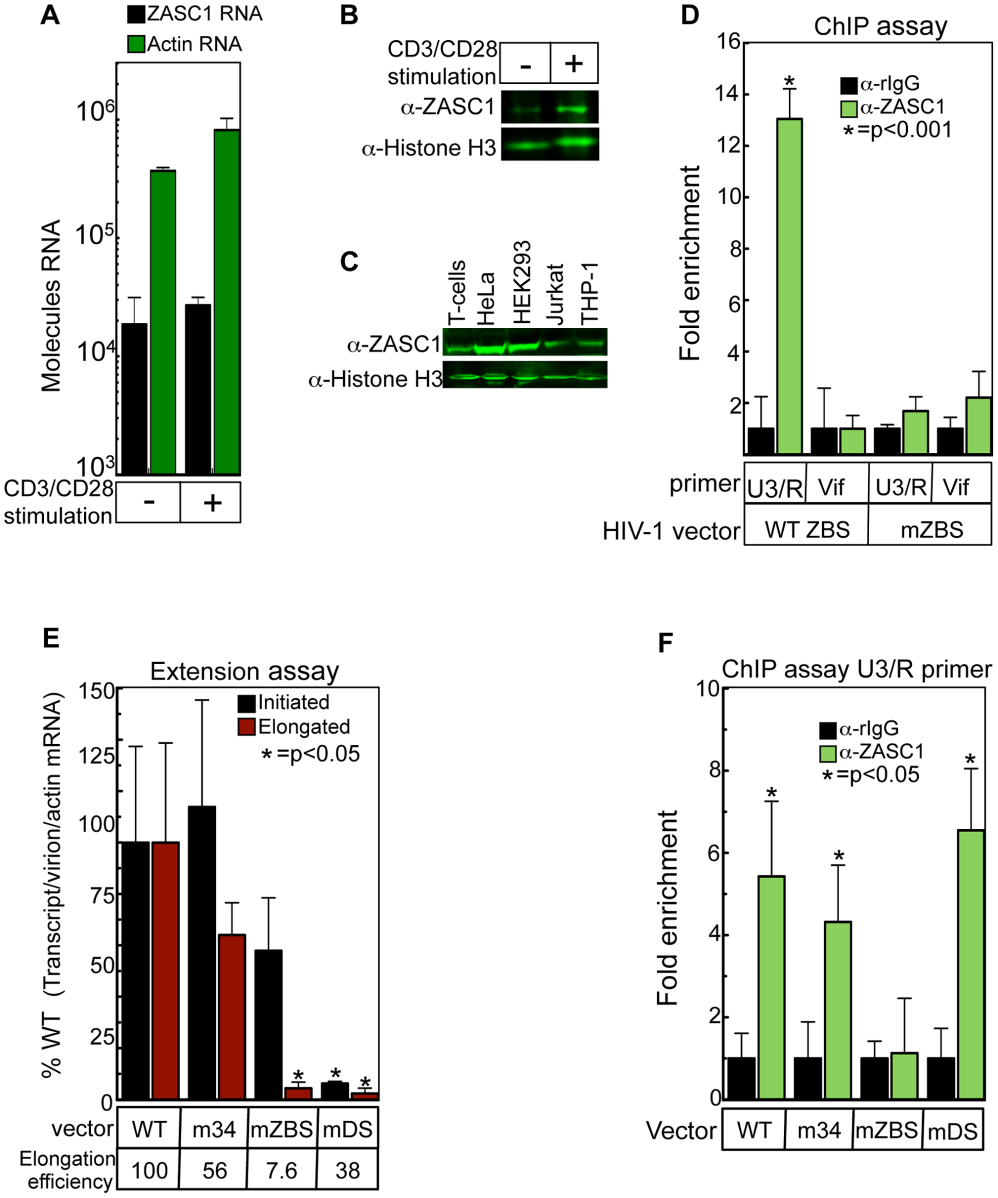
ZASC1 stimulates transcription elongation from the HIV-1 promoter in primary T-cells. (A) Isolated CD4+ were placed in culture and stimulated for 24 hrs with CD3/CD28 beads, total RNA was isolated from cells (1×10^5^) and expression of ZASC1 and actin was monitored by quantitative real-time RT-PCR. (B) Unstimulated or CD3/CD28 stimulated primary T-cells (1×10^6^) were harvested and analyzed by immunoblotting with anti-ZASC1 and anti-Histone H3 antibodies. (C) Cells (1×10^6^) from CD3/CD28 stimulated primary T-cells or the indicated cell lines were harvested and analyzed by immunoblotting with anti-ZASC1 and anti-Histone H3 antibodies. A representative blot of three independent experiments is shown (D) Stimulated primary T cells were challenged with either WT NL43-luc or mZBS mutant and 2 dpi, total ZASC1 bound to the HIV promoter was measured by chromatin immunoprecipitation with an anti-ZASC1 antibody using a primer set that spans the ZBS at the U3/R boundary (−116 to +25) or a primer that amplifies the vif locus located approximately 5 kBp downstream from the transcription start site. Real-time PCR analysis was performed in triplicate and normalized to input controls and fold enrichment relative to rabbit IgG controls reported. (E) The amount of initiated and extended transcripts was assayed in stimulated primary T-cells challenged with NL43E-R-Luc or ZBS variants by isolating total RNA 48 hpi and performing real-time reverse transcription as described in Fig. 3B. Transcript values were normalized for the amount of input virion RNA and total cellular actin mRNA. Real-time PCR was performed in triplicate. Elongation efficiency was determined by dividing the extended transcripts by the total initiated transcripts with WT efficiency set at 100%. (F) Stimulated primary T-cells were challenged with the indicated NL43-Luc variants and 2 dpi total ZASC1 bound to the HIV-1 promoter was determined by ChIP analysis as in C. Error bars indicate the standard deviation of the data and are representative of three independent experiments. P-values were calculated using a standard Student's t-test.

The above data demonstrated that ZASC1 regulates HIV-1 transcription elongation in established cell lines. We next investigated if ZASC1 regulates HIV-1 transcription elongation in primary T-cells. Quantitative real-time RT-PCR showed that ZASC1 mRNA was expressed to similar amounts in both stimulated and unstimulated primary T-cells (Fig. 4A), although a modest, 2.6-fold increase in ZASC1 protein levels after stimulation was observed (Fig. 4B). Western blot analysis showed that the amount of ZASC1 in stimulated primary T-cells is comparable to that in the Jurkat T-cell line and the THP-1 monocyte lines commonly used in transcription studies (Fig. 4C). HeLa and HEK293 cell lines express more ZASC1 (6.7- and 4.4-fold, respectively) than primary T-cells (Fig. 4C). ChIP assays of primary T-cell chromatin with an anti-ZASC1 antibody enriched the U3/R boundary region (nt −116 to +25) of the HIV-1 promoter 13-fold, relative to a non-specific IgG control (Fig. 4D). This is an even greater enrichment than we observed in either Jurkat or HEK293 cell lines (Fig. 1C). Consistent with our previous results, no enrichment with the anti-ZASC1 antibody was observed with the mZBS virus or with primer sets that amplified downstream of the transcription start site with either virus (Fig. 4D). These data confirm that ZASC1 is recruited to the HIV-1 promoter in primary T-cells.

To determine if ZASC1 recruitment contributes to transcription elongation in primary T-cells, we assayed for initiated and extended transcripts in RNA isolated from primary T-cells that had been challenged with near equivalent amounts (within 2-fold) of WT NL43-luc, mTA, mZBS, and a control, downstream mutation mDS (See Fig. 1A). Similar to the results in cell lines, the mZBS variant exhibited modest defects (48%) in transcription initiation but a 23-fold defect in elongation (Fig. 4E). When corrected for the slight initiation defect, this results in an elongation efficiency of 13-fold. Importantly, a ZBS m34 mutation immediately upstream of the critical ZBS12 site exhibited no significant defect (Fig. 4E) in either elongation or initiation. A mutation located downstream of ZBS12 (mDS, Fig. 1A) exhibited a strong, 16-fold initiation defect (Fig. 4E). A slight, additional 2.6-fold defect in elongation was also detected in this mutation resulting in an elongation efficiency of 38% of WT (Fig. 4E). Thus demonstrating that our assay can detect multiple initiation and extension phenotypes and all perturbations of the core promoter do not result in the same elongation defect. Furthermore, the elongation defect tracked with ZASC1 recruitment to the HIV-1 LTR, with only the mZBS mutation exhibiting loss of ZASC1 binding (Fig. 4F). Taken together, these data demonstrate that both in cell lines and primary human T-cells, ZASC1 is recruited to the HIV-1 promoter and regulates transcription elongation.

### ZASC1 does not affect the basal activity of the HIV-1 promoter

**Figure 5:**
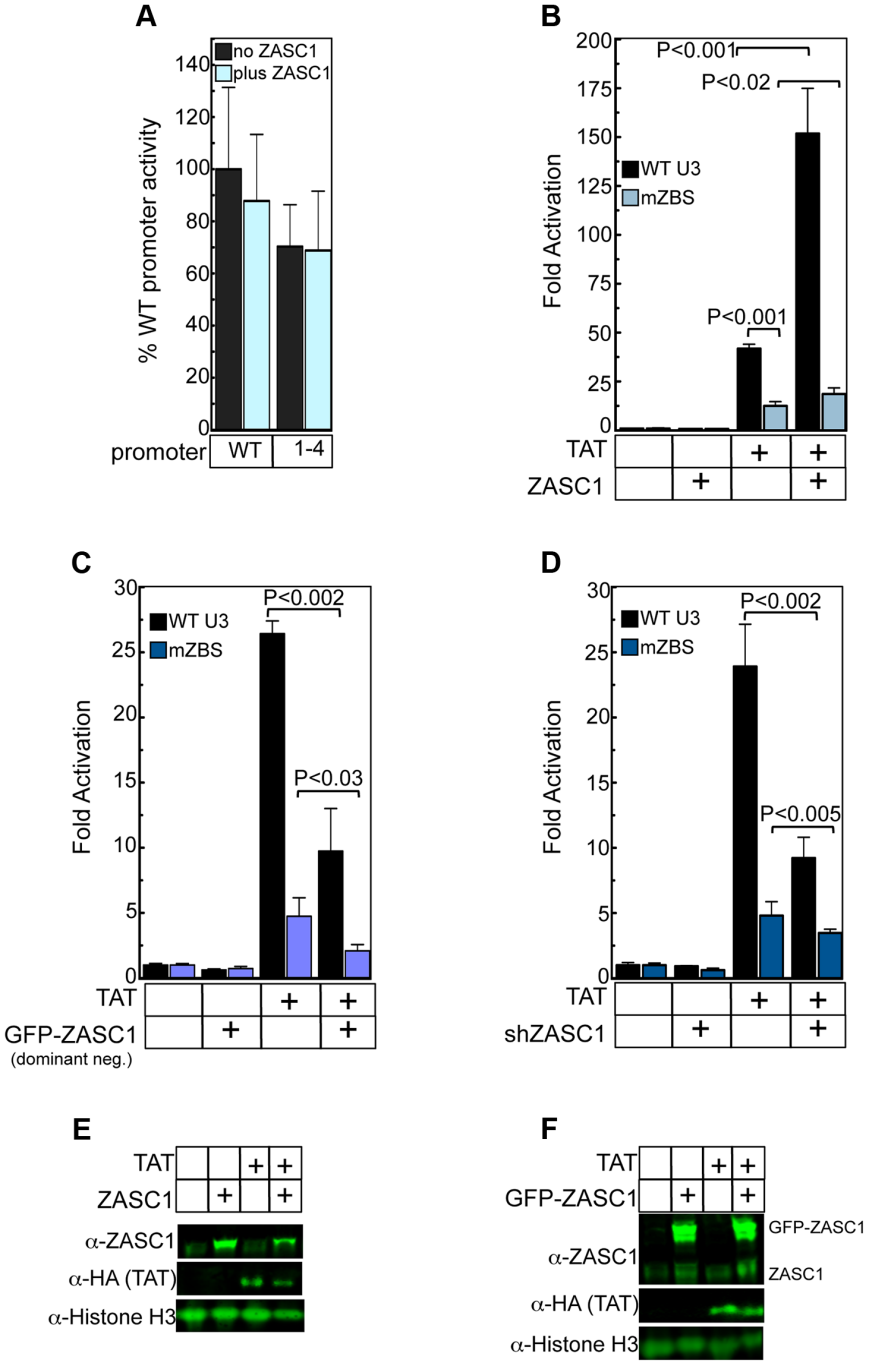
ZASC1 stimulates TAT-activation of the HIV-1 promoter. (A) HEK293 cells transiently transfected with a plasmid that expresses *Guassia* luciferase under the regulation of either the WT or mZBS 1–4 HIV promoter (all 4 putative ZBS mutated as in Fig. 1A) transfected in the presence of a ZASC1 expression plasmid. The basal activities of the WT and mZBS HIV promoters in the absence of ZASC1 were set to 100%. (B) Transient transfection of WT or mZBS HIV-1 promoters in the presence of plasmids that express TAT, ZASC1, or both TAT and ZASC1. (C) Transient transfection of WT or mZBS HIV-1 promoters in the presence of plasmids that express TAT, dominant negative GFP-ZASC1, or both TAT and GFP-ZASC1. (D) Transient transfection of WT or mZBS HIV-1 promoters in the presence of plasmids that express TAT, an shRNA plasmid that targets ZASC1 (shZASC1#4 see Fig. 2B), or both TAT and shZASC1#4. Fold activation by TAT and ZASC1 on the WT and mZBS promoters was determined relative to promoter expression in the presence of an empty expression plasmid. The transfection data shown are the average mean values obtained in an experiment performed with quadruplicate samples and each is representative of three independent experiments. Error bars indicate the standard deviation of the data in all panels. P-values were calculated using a standard Student's t-test and significant changes relative to WT or relevant bracketed comparisons indicated. Western blot of ZASC1, TAT and Histone H3 levels in HEK293 cells transiently transfected with plasmids that express (E) express TAT, ZASC1, or both TAT and ZASC1 or (F) TAT, dominant negative GFP-ZASC1, or both TAT and GFP-ZASC1.

To test the effects of mutating the ZBS on the basal activity of the HIV-1 promoter, HEK293 cells were transfected with a reporter plasmid that expressed *Gaussia* luciferase under the control of either the WT HIV-1 promoter, or this promoter with all four putative ZBS mutated (mZBS) as described in figure 1A. These constructs lack the HIV-1 transcriptional regulator TAT, thereby allowing us to measure the effect of the promoter mutations on basal HIV-1 promoter activity. In striking contrast to the results obtained from infection by a full HIV-1 vector (Fig. 2A), the basal activity of the HIV-1 mZBS promoter in this plasmid context was not significantly different from the WT promoter, and neither WT nor the mZBS promoter was stimulated by co-transfecting a ZASC1 expression plasmid (Fig. 5A). This is distinct from our previous results with the MLV promoter, where mutating the ZBS reduced the basal promoter activity 3-fold and expressing ZASC1 stimulated the promoter activity [27]. These studies indicate that the viral TAT protein may be important for ZASC1-dependent regulation of HIV-1 transcription.

### ZASC1 stimulates TAT activation of the HIV-1 promoter

To directly test whether ZASC1 influences HIV-1 TAT-mediated transactivation, plasmid constructs bearing WT or mZBS HIV-1 promoters were co-transfected with plasmids encoding either ZASC1, TAT or both. As before (Fig. 5A), ZASC1 overexpression had no effect on the basal activity of either the WT or mZBS promoter in the absence of TAT (Fig. 5B). Expression of TAT alone stimulated expression from the WT HIV-1 promoter by 42-fold, but by only 10.5-fold from the mutant mZBS promoter (Fig. 5B), showing that the ZASC1 DNA binding sequence contributes to TAT-mediated transactivation, presumably by recruiting endogenous ZASC1. Furthermore, the WT promoter activity was enhanced an additional 3.6-fold when TAT was co-expressed with ZASC1 (Fig. 5B). In contrast, there was little effect of co-expressing ZASC1 with TAT on expression from the mutant mZBS viral promoter (Fig. 5B).

As previously shown (Fig. 4C), HEK293 cells have a significant amount of endogenous ZASC1. We tested if endogenous ZASC1 contributes to TAT activation by transfecting cells with either an expression construct encoding the dominant negative GFP-ZASC1 fusion protein or a ZASC1 shRNA plasmid (shRNA ZASC#4, Fig. 2B). As with WT ZASC1, neither of these constructs significantly affected basal activity of the HIV-1 promoter (Fig. 5C and 5D). However, both dominant-negative GFP-ZASC1 fusion protein and ZASC1 knockdown significantly reduced TAT activation of the WT HIV-1 promoter (26- to 9.7-fold and 24-to 9.2- fold, respectively) (Fig. 5C and 5D). As expected, transfection of the ZASC1 shRNA#4 construct had no effect on TAT activation of the mZBS promoter (Fig. 5D). However, the dominant negative ZASC1 did show a slight but significant reduction in TAT activation of the mZBS promoter. Since the dominant negative ZASC1 did not affect TAT expression levels (Fig. 5F), it seems likely that the dominant negative ZASC1 is affecting the mZBS promoter through another mechanism that does not require the HIV-1 ZBS, such as sequestering required co-factors at promoters with a functional ZBS. To rule out that ZASC1 overexpression was affecting TAT accumulation, western blot analysis was performed. ZASC1 overexpression did not increase TAT accumulation and thus account for the increased TAT activation (Fig. 5E), nor did GFP-ZASC1 negatively affect TAT accumulation (Fig. 5F). Thus, ZASC1 contributes to TAT-mediated activation of the HIV-1 gene expression, and does so primarily through the ZASC1 binding sites in the promoter.

### ZASC1 interacts with HIV-1 TAT and cellular P-TEFb

**Figure 6:**
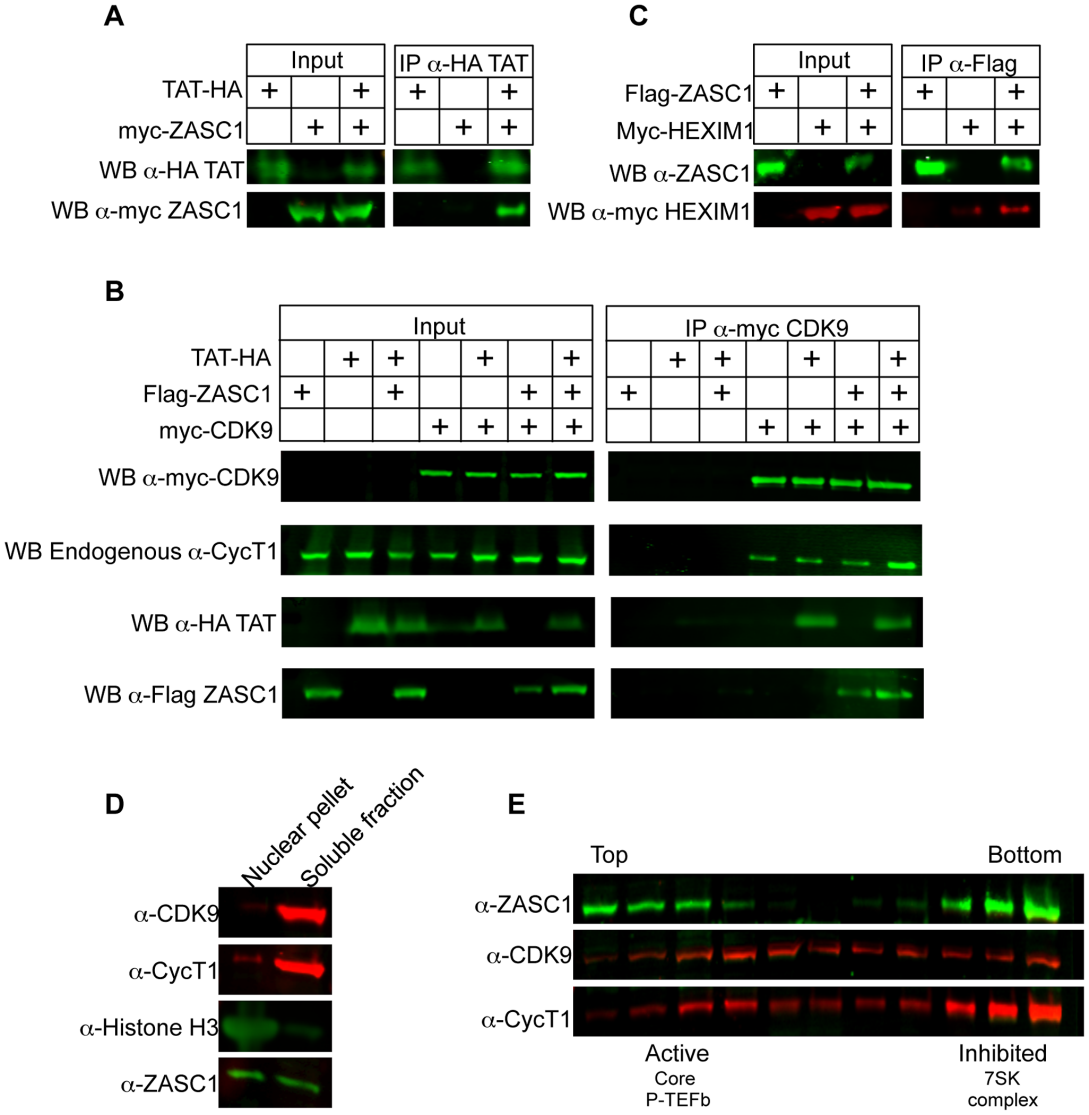
ZASC1 binds TAT and P-TEFb. HEK293 cells (1×10^7^) were transfected with expression plasmids encoding the epitope tagged forms of the indicated proteins. 48 h post-transfection, cells were lysed and epitope tagged proteins were immunoprecipitated (IP), separated by SDS-PAGE and analyzed by western blotting (WB) using the indicated antibodies as described in materials and methods. (A) Co-immunoprecipitation of Flag-ZASC1, and HA -TAT following IP with anti-HA beads.(B) Co-immunoprecipitation of HA-TAT, Flag- ZASC1 and Myc-tagged CDK9 following IP with anti-Myc beads. (C) Co-immunoprecipitation myc-Hexim1 and Flag-tagged ZASC1 following IP with anti-Flag beads. (D) Western blots of HEK293 cell fractionation into nuclear pellet and soluble fractions before glycerol gradient analysis. (E) Western blot of fractions from a 5% to 45% glycerol gradient of HEK293 cell lysates. All blots are representative of at least three independent experiments.

To determine if ZASC1 associates with TAT and with components of P-TEFb, we performed co-immunoprecipitation experiments using epitope-tagged proteins. Micrococcal nuclease was included in all immunoprecipitations to preclude the effects of nucleic acids on the interactions. Precipitation of HA-tagged TAT resulted in co-purification of Myc-tagged ZASC1 (Fig. 6A). To determine if ZASC1 complexes with P-TEFb in cells, Myc-tagged CDK9 was co-expressed with FLAG-tagged ZASC1 and HA-tagged TAT. FLAG-tagged ZASC1 co-immunoprecipitated with CDK9 in the presence or absence of TAT (Fig. 6B), suggesting that P-TEFb is a natural interaction partner of ZASC1. Endogenous cyclinT1 could also be detected in these ZASC1/CDK9 co-immunoprecipitations (Fig. 6B). Thus, ZASC1 associates with TAT and, in a TAT-independent manner, with P-TEFb within cultured cells. These findings are consistent with a role for the associated TAT/P-TEFb complex in the transcriptional elongation that is promoted by ZASC1.

### ZASC1 associates with both inactive and active forms of P-TEFb

P-TEFb exists in cells as a free, active form and the inhibited 7SK snRNP bound form. We detected a weak co-immunoprecipitation of ZASC1 with HEXIM1 (Fig. 6C), a component of the inhibitory 7SK snRNP complex, suggesting a role for ZASC1 recruiting the inhibited form of P-TEFb to the promoter. Cell lysates were fractionated on glycerol gradients to determine which form of P-TEFb ZASC1 interacts with. Under standard glycerol gradient conditions in which P-TEFb components are primarily in the soluble fraction, ZASC1 is evenly split between the DNA containing nuclear pellet and the soluble fraction (Fig. 6D). The soluble fraction of ZASC1 fractionated into two distinct peaks on glycerol gradients that correlated well with the heavy (inhibited) and light (active) forms of P-TEFb (Fig. 6E) [8], [10] suggesting that ZASC1 can associate with both forms of P-TEFb.

**Figure 7:**
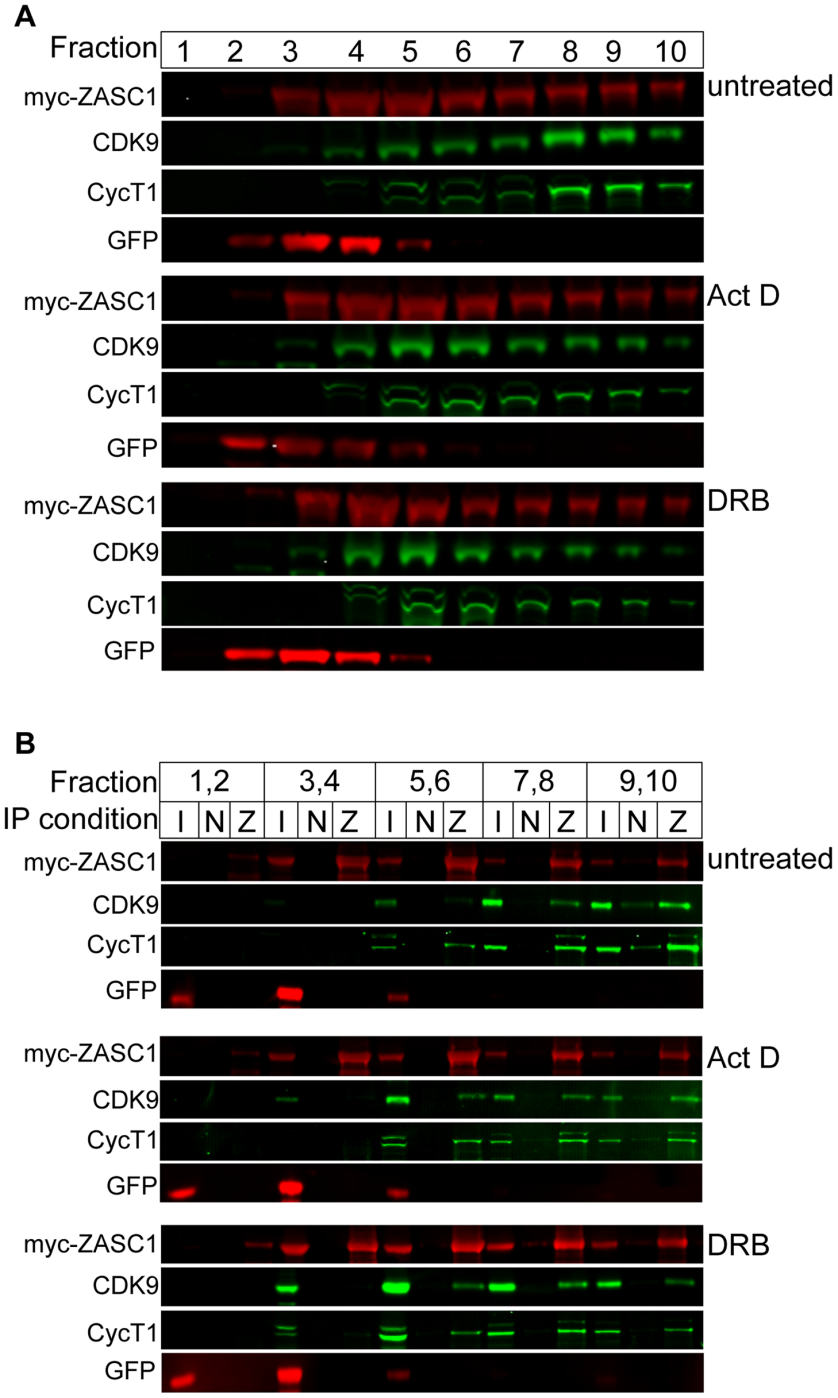
ZASC1 binds to active and inactive forms of P-TEFb. HEK293 cells (1×10^8^) were transfected with expression plasmids encoding Myc-tagged ZASC1 and eGFP. 48 h post-transfection, cells were treated with 10 µM DRB or 1 µg/ml actinomycin D for one h, lysed, fractionated on a 5% to 45% glycerol gradient and (A) analyzed by western blot with the indicated antibodies. (B) The indicated gradient fractions were pooled and immunoprecipitated with either a non-specific mouse IgG or mouse anti-Myc IgG. Input (I) non-specific (N) and myc-ZASC1 (Z) immunoprecipitations were analyzed by western blot with the indicated antibodies. All blots are representative of at least three independent experiments.

To confirm whether ZASC1 is able to interact with both the 7SK shRNP bound and unbound forms of P-TEFb, we performed glycerol gradient sedimentation followed by co-immunoprecipitation. HEK293 cells were transfected with Myc-tagged ZASC1 and eGFP expression plasmids. Unlike endogenous ZASC1, which exhibited a fairly even split between the top and bottom of the gradient (Fig. 6E), overexpression resulted in a fairly even distribution of ZASC1 between fractions 3 and 10, with peak fractions between 4 and 6 (Fig. 7A). In contrast, expressed eGFP migrated primarily at the top of the gradient in fractions 3 and 4. P-TEFb components localized primarily to peak fractions 8–10, with lesser amounts distributed through fractions 4 to 7. Treatment of cells with the transcription inhibitors 5,6-dichloro-1-β-D-ribofuranosylbenzimidazole (DRB) or actinomycin D (ActD), which causes the activation of P-TEFb [39], resulted in the shift of CycT1 and CDK9 to fractions 4 to 6. These treatments had no effect on the level or distribution of the transfected ZASC1 and eGFP proteins (Fig. 7A). Gradient fractions were pooled in pairs and immunoprecipitated with either a non-specific IgG or anti-Myc antibody (Fig. 7B). In untreated cells, P-TEFb components CDK9 and CycT1 co-immunoprecipitated with ZASC1 in fractions 7,8 and 9,10, corresponding to the inhibited, 7SK-bound form, but faint co-immunoprecipitation also was observed in fractions 5,6 corresponding to 7SK-free, active P-TEFb. After treatment with ActD or DRB, in keeping with the increase in the 7SK-free, active form of P-TEFb (Fig. 7A, fractions 5–6), immunoprecipitation revealed an increase in ZASC1 interaction with active P-TEFb (Fig. 7B, pooled fractions 5,6). No ZASC1 interaction with eGFP was observed in treated or untreated cells (Fig. 7B). Taken together, these data imply that ZASC1 interacts with both the active and inactive forms of P-TEFb. However, since a significant proportion of ZASC1 remains associated with DNA under these conditions, it is critical to determine what is occurring at the HIV-1 promoter.

### ZASC1 mediates TAR-independent recruitment of TAT and P-TEFb to the HIV-1 promoter

**Figure 8:**
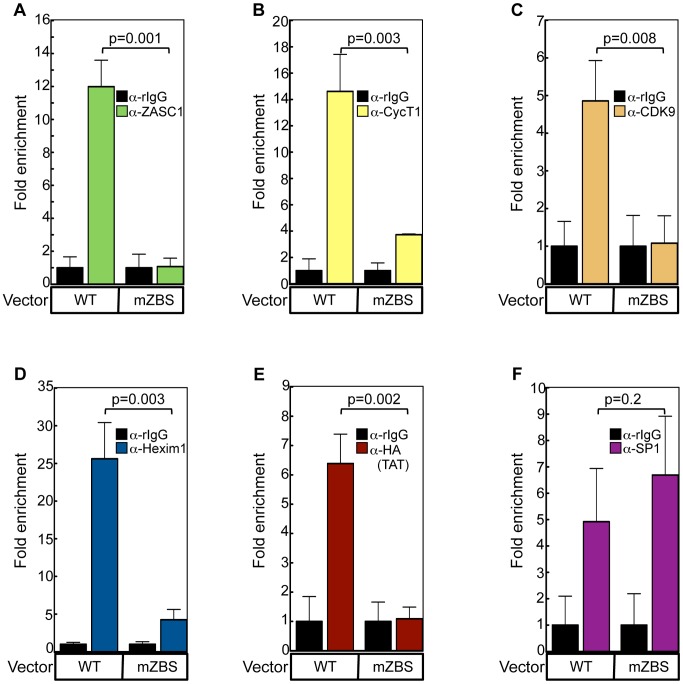
ZASC1 recruits P-TEFb and TAT to the HIV-1 promoter in the absence of TAR. The ability of ZASC1 to recruit P-TEFb and TAT to the HIV promoter was assessed by stably transfecting HeLa cells with a reporter plasmid containing a HIV promoter lacking a TAR element (WT or a variant with all ZBS mutated) and driving expression of the gLUC reporter enzyme. For TAT ChIP, the cells were transfected with HA-tagged TAT. ChIP experiments against endogenous proteins or TAT were performed using antibodies against (A) ZASC1 (B) CycT1 (C) CDK9 (D) Hexim1 (E) HA epitope (TAT) and (F) SP1. Real-time PCR was performed in triplicate using a primer set that spans the ZBS at the U3/R boundary (−116 to +25). Error bars indicate the standard deviation of the data and are representative of three independent experiments. P-values were calculated using a standard Student's t-test.

We next tested whether ZASC1 is involved in recruitment of the TAT/P-TEFb complex to the HIV-1 core promoter. To eliminate the effects of TAR on recruitment, we generated stable cell lines with a WT or mZBS HIV-1 promoter lacking the TAR element. We then assayed for recruitment of P-TEFb and TAT using ChIP assays, under conditions where the U3/R boundary of the WT HIV-1 promoter showed a 12-fold enrichment of ZASC1, relative to control immunoprecipitations, but there was no observable ZASC1 enrichment with a variant with mutated ZBS1-4 (Fig. 8A). Under these conditions, P-TEFb components cyclin T1 (Fig. 8B), CDK9 (Fig. 8C), Hexim1 (Fig. 8D) as well as TAT (Fig. 8E) were efficiently recruited to the WT, but not to the mutant, HIV-1 promoter. Thus, consistent with our IP and glycerol gradient results, ZASC1 can likely interact with both the active and inactive components of P-TEFb. Importantly, there was no defect seen with recruitment to the altered mZBS HIV-1 promoter of cellular transcription factor SP1 (Fig. 8F). SP1, which binds immediately upstream of ZBS1-3 (Fig. 1A), is critical for transcription initiation from the HIV-1 promoter [40], [41], and has been implicated in facilitating TAT function [42], [43], [44], [45]. The unaltered SP1 binding (Fig. 8F) implies that the observed defects in TAT and P-TEFb recruitment (Fig. 8B–E) must reflect recruitment that either is independent of SP1, or requires both ZASC1 and SP1. Thus, ZASC1 is a TAR-independent mediator of TAT and P-TEFb recruitment to the HIV-1 core promoter, enhancing HIV-1 transcription elongation.

## Discussion

### ZASC1 is a novel activator of HIV-1 transcription

Here we have provided multiple, complementary lines of evidence that ZASC1 is a sequence-specific DNA binding protein that that stimulates transcription elongation from the HIV-1 LTR promoter by TAR-independent recruitment of TAT and P-TEFb in both standard laboratory tissue culture cell lines and in primary T-cells. Previously, we showed that ZASC1 regulated MLV transcription through interaction with three binding sites in the MLV U3 promoter [27]. Sequence analysis of the HIV-1 promoter revealed four potential ZBS in the HIV-1 promoter (Fig. 1A), and we demonstrated that ZASC1 binds to this promoter in vitro (Fig. 1B) and in vivo (Fig. 1C&D).

Mutation analysis revealed that ZASC1 binding to two overlapping anti-parallel binding sites (ZBS1 and ZBS2 in Fig. 1A & 2A), which forms a 2 bp offset inverted palindrome, partially overlaps the TATA box and is positioned between the TATA box and the +1 site of transcription (Fig. 1A) severely reduces HIV vector gene expression in Jurkat cells (Fig. 2A) and primary human T-cells (Fig. 3). Furthermore, shRNAs targeting ZASC1 as well as dominant negative forms of ZASC1 inhibit HIV-1 expression in a ZBS-dependent manner (Fig. 2C & D). Since the defect in these cells is due to ZASC1 depletion or dominant negative interference, the lack of a further reduction in gene expression in vectors with ZBS mutations demonstrate that these mutations specifically affect ZASC1 binding and do not perturb the function of other transcription factor binding sites.

**Figure 9:**
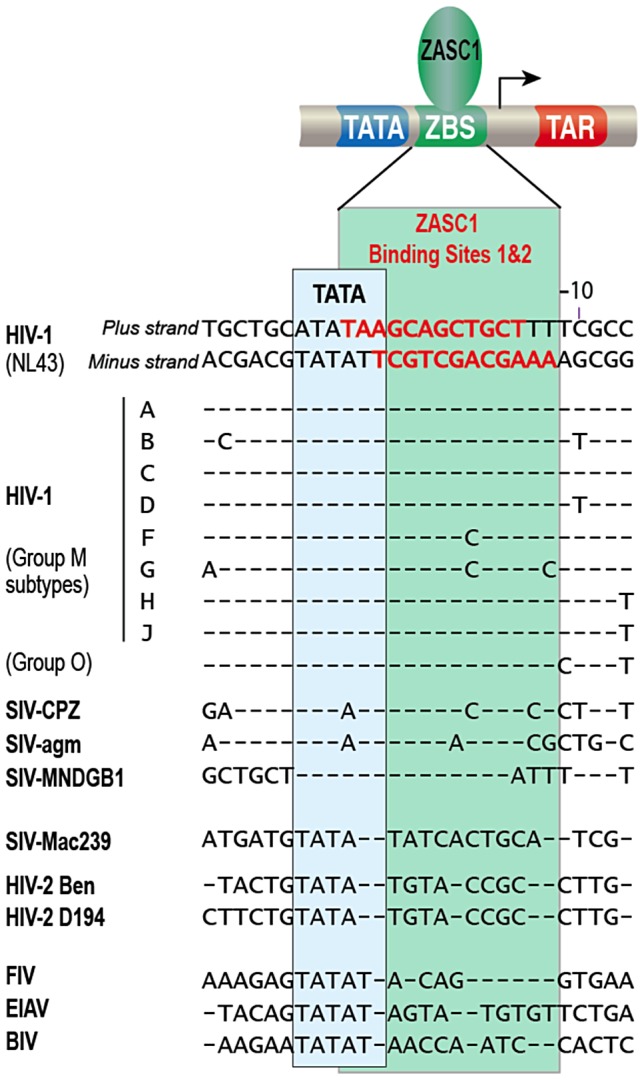
Alignment of ZBS1,2 binding sites. HIV-1 sequence from −7 to −34 from circulating HIV-1 strains from major and outlier clades, simian immunodeficiency viruses from Chimpanzee, African green monkey, Mandrill, Macaque, HIV-2, feline immunodeficiency virus, equine infectious anemia virus and bovine immunodeficiency virus were aligned to the HIV-1 NL43 strain used in this study as described in materials and methods. Conserved sequence shown as dashes, changes from NL43 are indicated.

Alignment of the HIV-1 promoter element around the TATA box with other lentiviral sequences reveals that this palindromic ZBS configuration is highly conserved across human HIV-1 clades and chimpanzee SIV isolates (Fig. 9), implying strong evolutionary pressure for its maintenance. At least one ZBS also is maintained in SIV isolates from mandarin and African green monkey. In contrast, human HIV-2 isolates as well as the HIV-2 like macaque isolate SIV-239 lack any conservation of ZBS sequences. It remains to be determined if this difference contributes to the reduced pathogenicity of HIV-2. These conserved ZASC1 binding sites were not found in the non-primate lentiviruses (Fig. 9)

The conserved region bearing the ZASC1 binding sites 1 and 2 partially overlaps binding sites for many other transcription factors, including TBP, LBP1, YY1, AP4 and RBF-2 [2], [46], [47]. RBF-2, a transcription factor composed of USF1, USF2 and TFII-I, binds within the RAS-responsive binding element I (RBEI) and RBEIII (Fig. 1A). Loss of RBF-2 binding at the upstream RBEIII, which overlaps ZBS4, has been shown to inhibit HIV-1 responsiveness to the Ras/MAP kinase signaling pathway but not to reduce basal or TAT-activated transcription [48], [49], [50], [51], [52], [53]. The effect of loss of RBF-2 binding to RBEI is less clear, but again is associated with loss of Ras/MAP kinase responsiveness and not of TAT-activated transcription [46], [48], an outcome that appears to be distinct from the roles of ZASC1 in HIV-1 transcription (see below). Indeed, the transcription factors previously ascribed to bind in and around ZBS1 and 2 sites have been associated with assembly of the pre-initiation complex and stimulation or inhibition of transcription initiation, and not TAT activated transcription elongation [46], [47], [48], [54], [55].

### ZASC1 modulates TAT-dependent activation of HIV-1 elongation

ZASC1 activates transcription from the MLV and HIV-1 promoters by distinct mechanisms. Mutating the ZBS in the MLV promoter reduces the basal activity of the promoter in transient transfection assays. In addition, co-transfecting ZASC1 stimulates the MLV promoter activity in a ZBS-dependent manner [27]. This suggests that ZASC1 functions as a classical transcriptional activator on the MLV promoter, possibly through recruiting P-TEFb (see below).

This mode of action is distinct from that of ZASC1 activation of the HIV-1 promoter. ZASC1 had little effect on the basal activity of the promoter, but severely inhibited TAT-mediated transcription activation (Fig. 5). Thus, ZASC1 activates the HIV-1 promoter in a TAT-dependent manner.

These conclusions are further supported by the observation that ZASC1 knockdown (Fig. 3C) had no significant effect on transcription initiation, but severely reduced transcription elongation. Consistent with this, the amount of initiated transcripts are relatively unaffected by mutating the ZBS's, and even minor ZBS mutations severely reduce the amount of elongated transcript generated from the HIV-1 promoter (Fig. 3B and Fig. 4E). Furthermore, recruitment of pol II to the site of transcription initiation is unchanged in ZBS mutants (Fig. 3D), but the presence of elongating pol II downstream of the promoter is strikingly impaired (Fig. 3E). Thus, ZASC1 plays a critical role in recruiting TAT and P-TEFb to the HIV-1 promoter and overcoming polymerase pausing.

### ZASC1 complexes with TAT and P-TEFb

Since ZASC1 activated the HIV-1 promoter through TAT, we asked if ZASC1 interacts with TAT (Fig. 6A). Co-immunoprecipitation demonstrated that ZASC1 complexes with TAT in cells. Additionally, ZASC1 co-immunoprecipitated with components of P-TEFb in the presence and absence of TAT (Fig. 6B). We have seen no evidence for competition between P-TEFb components and ZASC1 or TAT, (Fig. 6B and data not shown), and there may be a slight improvement in immunoprecipitation efficiency when P-TEFb, ZASC1 and TAT are co-expressed (Fig. 6B). However, since TAT is not required for the ZASC1:P-TEFb interaction, these data demonstrate that ZASC1 is a natural TAT-independent interaction partner of P-TEFb, and may function to recruit P-TEFb and TAT to the site of stalled transcription during HIV-1 infection. Moreover, these data suggest a role for ZASC1 in transcription elongation of TAT-independent viral and cellular promoters. Of particular interest is weather ZASC1 interacts with the active form of P-TEFb or the 7SK inhibited form of P-TEFb. We see an interaction between ZASC1 and the HEXIM1 component of the 7SK snRNP (Fig. 6C), albeit less robust then the interaction with CDK9 (Fig. 6D) or CycT1 (data not shown). Consistent with this, ZASC1 co-sediments with both the active and inactive forms of P-TEFb on a glycerol gradient (Fig. 6E, Fig. 7), implying that ZASC1 may interact with both forms of P-TEFb.

Since only half of ZASC1 is solubilized under glycerol gradient conditions, it is essential to determine what occur with the DNA-associated, presumably functional, ZASC1. ChIP analysis revealed that loss of ZASC1 binding correlates with a failure to recruit P-TEFb components and HEXIM1 (Fig. 8). Taken together, these data suggest that ZASC1 may recruit the inactive form of the complex but remain associated with P-TEFb for some time after activation.

Once P-TEFb is released from the 7SK snRNP, P-TEFb and TAT are transferred to the extending polymerase with TAT facilitating the recruitment of the superelongation (SEC) complex to the extending polymerase [5], [56], [57]. Consistent with an early role in P-TEFb recruitment, we have been unable to detect any interaction between ZASC1 the SEC components ELL2, AF4 and AFF4 (data not shown).

Our findings are supported by recent independent reports that full TAT activity requires sequences in the HIV-1 core promoter flanking the TATA box [16], [17], [18], [19], [20], [21], [22]. Interestingly, it was recently observed that CTGC motifs in the HIV promoter affect TAT function [23]. Since these critical motifs overlap ZBS1, 2 and 3, these observations are consistent with a role of ZASC1 in TAT recruitment. In addition to the role of the core promoter in TAT recruitment, evidence exists for either a direct or indirect interaction between the upstream binding SP1 transcription factor and TAT [42], [43], [44], [45]. However, SP1 also is essential for transcription initiation from the HIV-1 promoter [40], [41], and TAT stimulates the phosphorylation and thus the initiation-promoting activity of SP1 [58]. Taken together these observations suggest that at least two DNA-dependent recruitment events are required for full TAT function, one SP1- dependent and one SP1-independent. Indeed, on a TAR-less promoter, mutation of ZBS results not only in the loss of ZASC1 binding, but also abrogates the recruitment of P-TEFb components and TAT (Fig. 8). Significantly, loss of ZASC1 binding had no effect on SP1 binding (Fig. 8F). Thus, SP1 may facilitate but is not sufficient for TAT recruitment and stimulation of transcription elongation.

**Figure 10:**
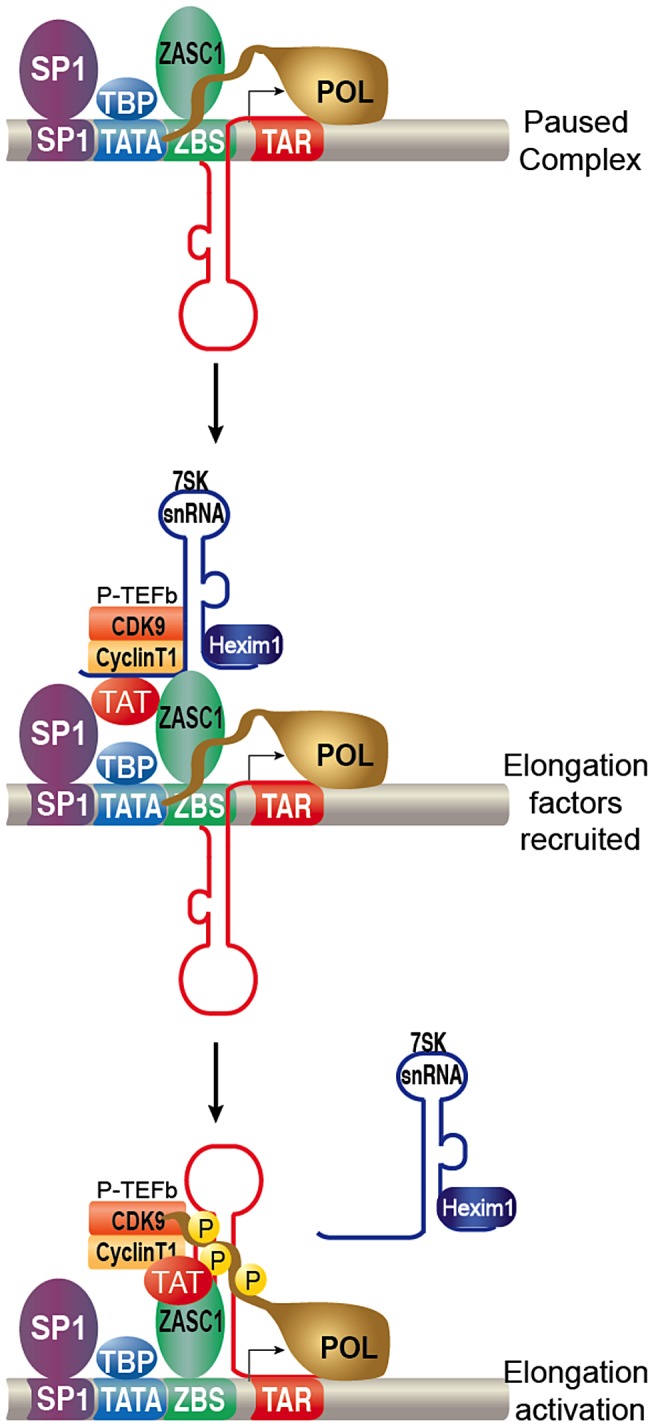
Model for ZASC1 function during HIV-1 transcription. Transcription efficiently initiates but the RNA pol II is non-processive. 7SK inhibited P-TEFb and TAT are recruited to the promoter by ZASC1. The complex undergoes a conformational change when TAT and TAR disassociate the inhibitory 7SK snRNA complex, TAT and P-TEFb transfers to TAR facilitating phosphorylation of the CTD of pol II and stimulating transcription elongation.

Taken together, these data further elucidate the mechanism by which HIV-1 overcomes the early block to elongation from the LTR promoter. Initially, transcription from the HIV-1 promoter efficiently initiates but rapidly stalls. Subsequently, ZASC1 binds downstream of the core promoter and recruits 7SK-inhibited P-TEFb and TAT. This high order complex which contains interactions between ZASC1, TAT, P-TEFb, 7SK snRNP and potentially SP1, may be required to allow the paused elongation complex to undergo a conformational change. Such a change may involve the TAR element competitively displacing the inhibitory 7SK snRNA [16], and is facilitated by TAT competing for the Hexim1 binding site on the 7SK snRNA [15] as well as the high affinity of TAT for CycT1 [11], [12], [13], [14]. Our data (Fig. 7B) imply that ZASC1 may remain associated with the active form of P-TEFb. The now active P-TEFb phosphorylates the pol II CTD, DSIF and NELF with P-TEFb and TAT subsequently transferred to the elongating polymerase (Fig. 10).

This model is especially attractive under conditions of low TAT concentrations such as early in infection or reactivation from latency. In resting T-cells, CycT1 levels are low while the levels of CDK9, in an inactive T-loop dephosphorylated state, are relatively constant. On activation, CycT1 levels increase, as does incorporation of P-TEFb into the 7SK-inhibited complex with TAT relieving this inhibition [59], [60], [61]. Thus ZASC1-recruiting inactive P-TEFb could facilitate efficient P-TEFb mobilization under low TAT conditions early after reactivation. Interestingly, while ZASC1 mRNA levels are not altered by T-cell activation (Fig. 4A) there is a slight increase in the protein levels of ZASC1 upon stimulation (Fig. 4B). Consistent with this, there is a report of ZASC1 being differentially phosphorylated after T-cell stimulation [61]. Accordingly, it will be important to investigate the role of post-transcriptional modifications on ZASC1 function and stability, both for HIV-1 infection and in T-cell biology.

Future work will be aimed at identifying the specific mechanisms by which ZASC1 binding regulates MLV and HIV-1 infection. Furthermore, we are currently investigating the cellular promoters regulated by ZASC1 in primary T-cells to further understand the role of ZASC1 in both T-cell biology and in the course of HIV-1 infection. We expect that this information will help to uncover precisely how cells regulate the transcriptional competency of the provirus and could lead to new therapies that either prevent the of reactivation of latent provirus, or stimulate reactivation and permit the subsequent clearing of latently infected cells in individuals on suppressive antiretroviral therapies.

## Materials and Methods

### Plasmids and viral vectors

The viral genome plasmids pHIV-TVA800-hcRED, pLenti6/V5-GW/lacZ, and pNL4-3.Luc.R-E- have been previously described [34], [62].

ZASC1 expression vectors were generated by PCR amplification of coding sequence (ZASC1(ZNF639):IMAGE#4794621) from commercially available cDNAs (Open Biosystems, Huntsville, AL) and cloning into pCMV-TNT (Promega, Madison, WI).

Melanie Ott kindly provided the plasmids pEV280, which expresses WT two-exon TAT with a C-terminal FLAG-tag from the HCMV promoter [63]. The plasmids pcDNA3 TAT-HA encoding two-exon HA tagged TAT expressed from the HCMV promoter were obtained from Matija Peterlin along with HA-tagged CDK9 and FLAG-tagged CycT1 expression plasmids [64] through Addgene Incorporated. Myc-tagged versions of CDK9 were made by PCR amplification of the CDK9 reading frame and cloning into pCMV-MYC (Clontech, Mountain View, CA

Short hairpin RNA (shRNA) constructs in the pcDNA6.2-GW/EmGFP-miR (Invitrogen, Carlsbad, CA) targeting ZASC1 or secreted alkaline phosphatase (SEAP) were previously described [27].

To generate retroviral promoters for EMSA assays and reporter gene analysis, the HIV-1 U3 and TAR element was amplified from pNL4-3.Luc.R-E- using the primers 5′-GGACGCGTTGGAAGGGCTAATTCACTCCC-3′ and 5′-GGAAGCTTAAGCAGTGGGTTCCCTAGTTAGC-3′. PCR products were digested with MluI and HindIII (underlined in primer sequence) and cloned into pGluc-Basic (NEB, Ipswich, MA). TAR deletion clones were generated by digesting the TAR containing clones with EcoRI and BglII and inserting the TAR-less promoter fragment into the EcoRI/BglII digested pGluc-Basic. Mutagenesis of potential binding sites was performed by overlap PCR or linker mutagenesis. HIV-1 genomic clones containing mutations in the ZBS were made by overlap PCR or linker mutagenesis on these subcloned fragments and moved back into the 3′LTR of full length pNL4-3.Luc.R-E- by standard restriction enzyme cloning. All mutations were validated by sequencing.

### Cell culture and virus production

The source and growth conditions human embryonic Kidney 293T cells, and human Jurkat cells were described elsewhere [62]. To generate the cell line expressing tetracycline inducible GFP-ZASC1, HeLa-TREX cells (Invitrogen, Carlsbad, CA) were transduced with pLenti4/TO-GFP-ZASC1. Transduced cells were selected with 100 µg/ml zeomycin and 5 µg/ml blasticidin. GFP-ZASC1 expression was induced with 1 µg/ml doxycycline. HeLa cells containing integrated plasmids with the HIV-promoter driving gLuc expression were generated by transfecting with pGluc-Basic derived constructs described above and selecting for in 400 µg/ml G418 for 14 days. The procedures used to produce the retroviral vectors and titer each viral stock are described in detail elsewhere [62].

Primary T-cells were obtained from Sanguine BioSciences (Santa Monica, CA), thawed into RPMI 1640 supplemented with 10% FBS and penicillin/streptomycin. Cells were stimulated with a 1∶1 ratio of anti-CD3 and anti-CD28 beads (Invitrogen, Carlsbad, CA) following manufacturer's instructions and expanded in the presence of 20 U/ml of IL-2 (Sigma, Saint Louis, MO). Cells were infected by spinoculation at 3000×g for 2 hrs in multi-well plates at an MOI of 3. Experiments with primary T-cells were repeated with cells from at least two different donors.

### Assays of viral infection

Quantitative chemiluminescent infection assays were performed as previously described [62], [65]. Briefly, 96 well plates were seeded at 1×10^4^ cells/well for each cell line tested. The cells were incubated with an approximate MOI of 1 transducing unit, 48 hpi, four wells were assayed for firefly luciferase (fLuc) activity using the Britelite reagent (PerkinElmer, Boston, MA) according to the manufacturer's instructions. The other four wells were assayed for cell number and cell viability using CellTiter-Glo reagent (Promega, Madison, WI). The results obtained were normalized for relative cell number.

### Electrophoretic mobility assays (EMSA)

ZASC1 EMSA conditions have been described in details elsewhere [27]. Briefly, 8 µl of an *in vitro* transcription/translation reaction was incubated at room temperature 10 minutes and on ice for 10 minutes with 50 fm ^[32]^P end labeled probe and 5 µl 5× EMSA buffer [250 mM Tris-HCl 7.5, 50 mM MgCl_2_, 25 ng/µl sheared CT DNA, 0.05% CHAPS, 5% glycerol, 10 µM Zinc acetate (Zn_2_(C_2_H_3_O_2_))] in a final volume of 25 µl. Bound DNA was separated from free probe on a 4% TBE polyacrylamide gel run at 125 V at 4°C for 2 hr. The gels were dried down and exposed to phosphorimager plates for analysis.

### Transient transfection assays

HEK293 cells (2×10^4^ cells/well) were reverse transfected in a 96 well format with a total of 100 ng DNA/well with 0.4 µl/well Transit LT-1 transfection reagent (Mirus, Madison) following manufacturer's instructions. For transient promoter activation assays, 10 ng of the retroviral reporter construct, 5 ng of a GFP expression plasmid, 5 ng of a firefly expression plasmid were included in all transfections. ZASC1 expression plasmids and TAT expression plasmids were included at 40 and 10 ng/well, respectively. Vector plasmid DNA or Calf thymus DNA was used to maintain a constant 100 ng/well in each well. Two days post-transfection, 10 µl of media was removed, diluted with 40 µl of PBS and assayed for secreted gaussia luciferase (gluc) by injecting 30 µl coelenterazine solution (*Renilla* luciferase assay system, Promega, Madison, WI), waiting 1.6 s and then reading luminescence for 1 s. Firefly luciferase (fLuc) activity from the internal control plasmid was determined using the Britelite (PerkinElmer, Boston, MA) according to the manufacturer's instructions. The activity of the retroviral promoter in each well was then expressed as the ratio of gLuc∶fLuc.

The retrovirus promoter plasmids consist of the HIV U3 promoter containing the TAR element, amplified and cloned in front of the secreted *Gaussia* luciferase reporter gene as described above. To monitor transfection efficiency, a plasmid (5 ng) encoding EGFP under the control of the HCMV promoter (pEGFP-C1, Clontech, Palo Alto, CA) and a plasmid (5 ng) encoding firefly luciferase under the control of the HCMV promoter were included in each transfection (5 ng). The plasmid encoding the ZASC1 open reading frame under the control of the HCMV promoter was obtained commercially (Openbiosystems, Huntsville, AL).

### Immunoprecipitation analysis

Cells (1×10^6^) were lysed in 500 µl ice cold Glycerol Gradient Lysis Buffer (GGLB) [150 mM NaCl, 10 mM KCl, 10 mM MgCl_2_, 10 mM HEPES (pH 7.5), 1 mM EDTA, 2 mM β-mercaptoethanol, 0.5% NP40, 1× ProteoBlock protease inhibitor cocktail (Fermentas, Hanover, MD)] buffer. Cells were allowed to lyse on ice for 10 min, and nuclei were pelleted at 20,000XG for 10 min, supernatant was transferred to a new tube and an aliquot was removed for SDS-PAGE. The remaining sample was immunoprecipitated with EZview Red Anti-HA, anti-FLAG affinity gels (Sigma, Saint Louis, MO), or anti-Myc agarose (Santa Cruz Biotechnology, Santa Cruz, CA) in the presence of 2,000 gel units micrococcal nuclease (NEB, Ipswich, MA) for 1 hour. The samples were washed three times in GGLB, and the beads resuspended in SDS-PAGE loading buffer. The samples were boiled, separated by SDS-PAGE, transferred to PVDF membrane and blotted with a rabbit polyclonal antibodies raised against either the HA epitope (Sigma, Saint Louis, MO), the FLAG epitope (Sigma, Saint Louis, MO), the Myc epitope (Santa Cruz Biotechnology, Santa Cruz, CA), human CDK9 (Santa Cruz Biotechnology, Santa Cruz, CA), human ZASC1 (Bethyl Laboratories, Montgomery, TX), human actin (Santa Cruz Biotechnology, sc-1616-R, Santa Cruz, CA) or GFP (Santa Cruz Biotechnology, Santa Cruz, CA). The blots were washed, treated with a secondary anti-rabbit horseradish peroxidase conjugate (Thermo scientific, Rockford, IL), and the blots imaged by either chemiluminesence using the Supersignal West femto substrate (Thermo scientific, Rockford, IL) or fluorescent antibodies (LI-COR Biosciences, Lincon, NE). Chemiluminescent signal was detected on a ChemiDoc XRS system (Bio-Rad, Hercules, CA). Fluorescent signal was detected on an Odyssey CLx (LI-COR Biosciences, Lincoln, NE). Virus release into media was detected using the monoclonal anti-p24 capsid antibody derived from the HIV-1 p24 Hybridoma (183-H12-5C) from Dr. Bruce Chesebro [66] obtained through the AIDS Research and Reference Reagent Program, Division of AIDS, NIAID, NIH. Reacting bands were visualized with anti-mouse horseradish peroxidase conjugate (Thermo scientific, Rockford, IL) following immunoblotting.

### Glycerol gradients

HEK293 cells (4×10^7^) were lysed and processed in GGLB as described above for immunoprecipitations. Supernatants were layered onto a 5% to 45% glycerol gradient made in GGLB. Gradients were spun at 190,000×g at 4°C for 16 hrs in a TLS-55 rotor. Fractions were analyzed by immunoblotting as described above.

### Chromatin immunoprecipitation (ChIP)

ZASC1 ChIP conditions were performed using a previously described [67] protocol with the following modifications. Formaldehyde crosslinked cells (2×10^7^) were sonicated for 40 cycles of 30 s on 30 s off in a Misonix Q700 cup horn Sonicator (Qsonica, Newtown, CT) at 95% power. Samples were incubated overnight with non-specific rabbit IgG (Millipore, Billerica, MA), rabbit anti-ZASC antibody A302-401A (Bethyl laboratories, Montgomery, TX), rabbit anti-SP1 A300-133A (Bethyl laboratories), rabbit anti-CDK9 SC-484 Santa Cruz Biotechnology, Santa Cruz, CA), rabbit anti-cyclinT1 SC-10750 (Santa Cruz Biotechnology), rabbit anti RNA polymerase II SC-899X (Santa Cruz Biotechnology), rabbit anti-Hexim1 SC- 134786 (Santa Cruz Biotechnology), or rabbit anti-HA SC-805 Santa Cruz Biotechnology). Immunecomplexes were purified with Protein A/G magnetic beads (Pierce, Rockford, IL). Immunoprecipitated DNA was analyzed by quantitative real-time PCR on a CFX96 (Bio-Rad, Hercules, CA) using SsoFast EvaGreen Supermix with low ROX following manufacturer's recommendations. Melt-curve analysis was performed in all assays to ensure product specificity and all assays were performed in triplicate. The primers used for analysis are described in Table 1.

### RNA extension analysis

Cells (2×10^5^) were infected with NL43 variants at an MOI of 3. Viral titer was determined by real-time reverse transcription PCR using the oJWB694/695 primers (Table 1). Only viral preps with titers within 2-fold of each other were used to inoculate cells for the extension assays. RNA was harvested 48 hpi using the miRNeasy kit (Qiagen, Valencia, CA) following manufactures instruction for isolation of long and short RNA species. Real-time reverse transcription PCR was performed on a CFX96 using iScript one-step Quantitative RT-PCR kit (Bio-Rad, Hercules, CA). Primers oJWB694/695 were used to amplify initiated transcripts. Either oJWB874/875 (luciferase gene in the nef locus) or oJWB872/872 (gag locus) were used to amplify extended transcripts. Primers oJWB887/888 were used to amplify cellular β-actin transcripts (Table 1). All analysis was performed in triplicate with melt curves to ensure product specificity. Results were normalized as viral transcript/input virion/actin mRNA.

### Sequence alignment

Sequences from major and outlier strains of HIV-1 (Genebank accession numbers M19921, AB253421, K03455, U52953, k03454, AF077336, AF084936, VI991, AF082394, L20571) Simian immunodeficiency virus (SIV) Chimpanzee (AF103818), SIV African green monkey (M29975), SIV Mandrill (M27470.1), HIV-2 (M30502, J04542), SIV Macaque 239 (M33262), feline immunodeficiency virus (M25381), Equine immunodeficiency virus (M16575) and bovine immunodeficiency virus (M32690) were aligned using the ClustalW program on the MacVector software package (MacVector Inc., Cary, NC).
